# Commentary: L-3-n-butylphthalide Rescues Hippocampal Synaptic Failure and Attenuates Neuropathology in Aged APP/PS1 Mouse Model of Alzheimer's Disease

**DOI:** 10.3389/fnagi.2017.00004

**Published:** 2017-01-26

**Authors:** Prakash C. Bhatt, Preeti Pandey, Bibhu P. Panda, Firoz Anwar, Vikas Kumar

**Affiliations:** ^1^Faculty of Pharmacy, Microbial and Pharmaceutical Biotechnology Laboratory, Centre for Advanced Research in Pharmaceutical ScienceJamia Hamdard, India; ^2^Department of Biochemistry, Faculty of Science, King Abdulaziz UniversityJeddah, Saudi Arabia; ^3^Natural Product Drug Discovery Laboratory, Department of Pharmaceutical Sciences, Faculty of Health Sciences, Sam Higginbottom University of Agriculture, Technology & SciencesAllahabad, India

**Keywords:** L-3-n-butylphthalide, neuropathology, APP/PS1 mouse, Alzheimer's disease, β-amyloid (Aβ)

According to World Health Organization estimation about 18 million people worldwide are suffering from Alzheimer's disease (AD) and figures would shoot up to 34 million by 2025 in aging population. The onset and propagation of AD is unrelenting from cellular to molecular levels, governed by multitude of factors leading to a continual decrease in cognitive abilities (Felsenstein et al., 2014). Recent advances in understanding the genetic factors that predispose to AD, as well as in biomarker development have brought with them the promise of earlier and more reliable diagnosis of this disease. However, treatment regime and strategies available, approved by US-FDA are limited by their scope to alter pharmacological targets that can provide symptomatic relief to the patient (Lannfelt et al., 2014). The present stock of drugs for AD consists of donepezil, galantamine, rivastigmine, and memantine. The primary mechanism of action for these drugs is to decrease the acetyl cholinesterase (AChE) levels thus altering acetylcholine level, while memantine is a low-affinity voltage-dependent uncompetitive antagonist at glutamatergic N-methyl d-aspartate receptor (NMDA) thereby blocking the activity of the neurotransmitter glutamate. All the existing treatments for AD are only symptomatic but non curative (Lannfelt et al., 2014). Under these circumstances a drug molecule with multiple pharmacological properties with intent to modify as well as reduce the risk factors contributing toward disease burden will be highly appreciated. Drugs that are aimed at disrupting AD disease progression by inhibition/degradation of the protein mis-folding of β-amyloid (Aβ) into neurotoxic oligomeric aggregates has been encouraged for AD (Scheff et al., 2007).

With the advent of new diagnostic tools various pathological conditions have been identified at different stages of AD. Accumulation of amyloid beta (Aβ) to form clumps known as neuritic or senile plaques is considered as hallmark of AD. The buildup of these plaques seems to be a crucial as they lead to oxidative stress coupled with mitochondrial dysfunction and barrage of inflammatory cascade causing neuronal cell death. Studies carried by Zhang et al. (2016) on L-3-n-butylphthalide (L-NBP) extracted from seeds of Chinese celery (*Apium graveolens* Linn) showed some promising results in animal models of AD. Interestingly treatment of L-NBP (15 mg/kg; oral, 3 months) to APP/PS1 mice resulted in amelioration of Aβ induced damage to synapse. Improvement in synaptic plasticity which is considered as an essential factor modulating cognitive functions in AD has also been observed after L-NBP. Pictorial results from electron microscopy revealed the significant increase in number of synapse and spines in hippocampus regions following treatment with L-NBP. It could be reasoned that multiple molecular events have been amended to bring about changes in synaptic strength. These changes might range from removal of synaptic plaques to anti oxidative and anti-inflammatory mechanisms. However, it is very hard to distinguish whether removal of synaptic plaques provides anti oxidative and anti-inflammatory relief or vice versa. In this study Zhang et al. (2016) explored pertinent proteins associated with synaptic plasticity in hippocampus to unveil the mechanism of neuroprotective effects of L-NBP and found upregulation in expression of PSD95, synaptophysin, β-catenin and GSK- 3β on treatment with L-NBP. PDS-95 is an abundant synaptic protein playing an important role in maintaining and regulating excitatory synapses and promoting synaptogenesis. β-catenin is a crucial factor for Wnt signaling pathway necessary for the maintenance of dendritic strength and density. An important issue remaining unanswered in these studies was involvement of adult neurogenesis following exposure to L-NBP. Since it affects multiple pharmacological targets it could be reasoned that L-NBP might alter stem cells in the hippocampal region to bring about changes in synaptic plasticity by involving the Wnt pathway. Whether the potential of L-NBP is limited to the degradation of Aβ or it might regulate proteins associated with adult neurogenesis via activation of stem cell markers in AD still remains elusive (Lei et al., 2014).

Interestingly, L-NBP influence on inflammatory responses during AD has been previously reported involving decreased expression of IBA-1 and inhibition of microglia activation in APP/PS1 mice. Furthermore, significant decrease in Aβ deposition linked with decreased gliosis has also been observed on after L-NBP exposure highlighting potential effects of the compound in ameliorating inflammation. Anti-inflammatory potential of L-NBP is well documented wherein it reduced neuro inflammation in lipopolysaccharide-treated C57BL/6 mice. The anti-inflammatory response could be beneficial in long term therapy in case of neurodegenerative disorders (Lei et al., 2014; Zhang et al., 2016).

Mitochondria are considered a target in neurodegenerative disorder since reactive oxygen species generated during ATP generation tip the balance toward neuronal damage and apoptosis (Figure 1). Elevated levels of mitochondrial ROS particularly in the neurons may target the lipid bilayers and cause neurochemical alterations. Further, compromised membrane potential could be associated with enhanced apoptosis due to activation of intrinsic apoptotic pathway. Amelioration of oxidative damage either through chelating ROS or promoting the expression of anti-oxidant enzymes is considered a potential mechanism through which L-NBP exerts its anti-oxidant effect (Peng et al., 2008, 2010).

**Figure 1:**
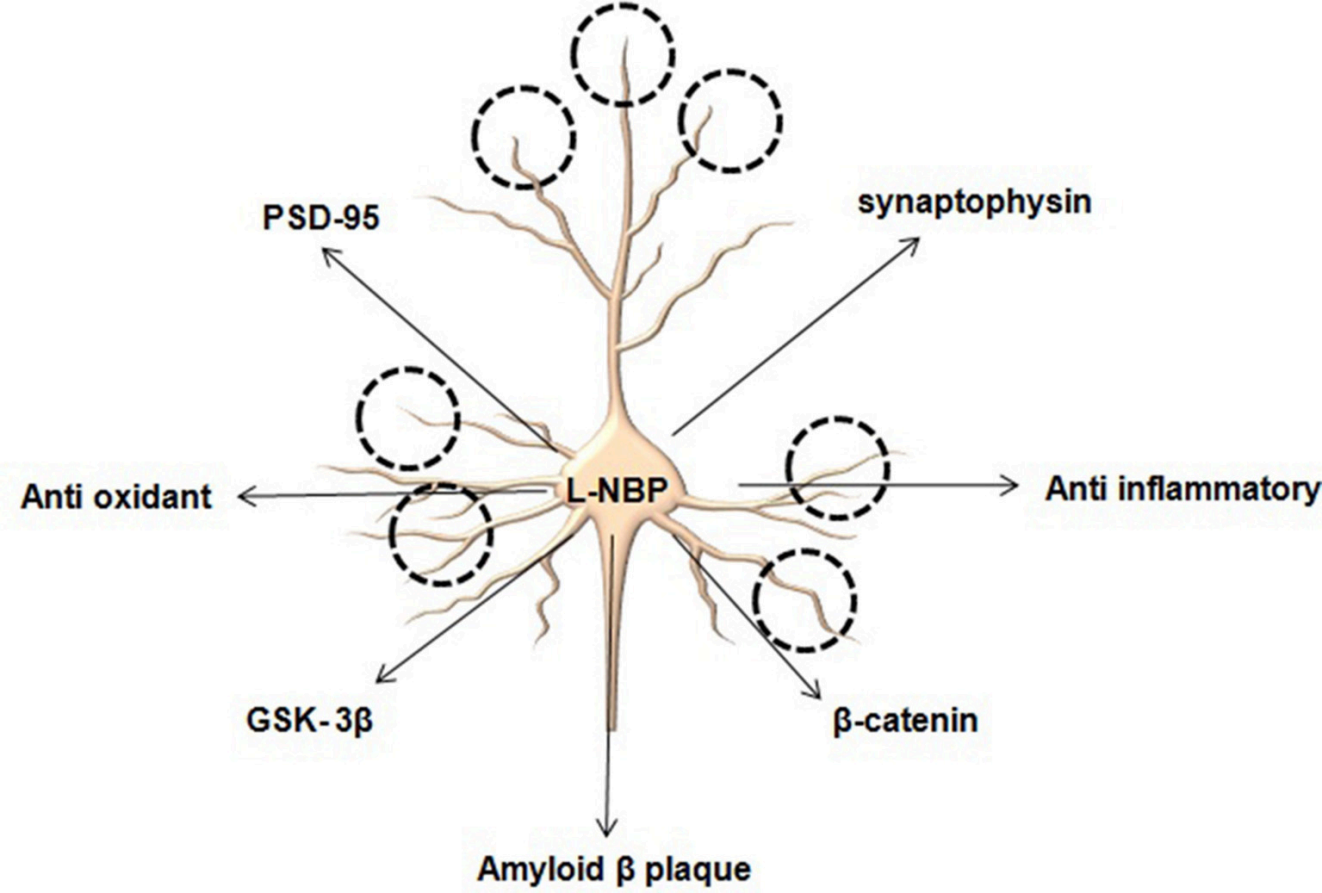
**L-NBP promotes synaptic number and synaptic strength by modulating several key proteins**.

Taken together, the findings suggest that the neurotherapeutic potential of L-NBP as a disease modifier in AD is due to its multiple pharmacological properties. It may be hypothesized from the experimental findings that the specific feature of this compound is the ability to reduce the load of Aβ and to exert anti-oxidant properties by lowering the levels of ROS, maintaining optimum MMP with reduction in inflammatory markers. For the first time Zhang et al. (2016) have demonstrated that this molecule could also repair synaptic damages via modulation of PSD95, synaptophysin (SYN), β-catenin, and GSK-3 β. L-NBP acts through various systems to impede progression of AD. Previous studies with L-NBP in patient with subcortical vascular cognitive impairment without dementia have demonstrated improved cognitive and global functioning and good safety (Jia et al., 2016). Another study by Xiang et al. (2014) also supported the neuroprotective role of L-BNP in an AD model by inhibiting oxidative injury, neuronal apoptosis and glial activation, regulating amyloid-beta protein precursor processing and reducing amyloid-beta formation. These studies substantiate the necessity of further evaluations of L-NBP for its potential beneficial effects in neurological diseases. However, the clinical efficacy of the molecule still requires to be evaluated. Bioavailability is considered as another major issue faced by natural compounds during clinical trials. Until disease-modifying therapies become available, further research is needed to develop new drug delivery strategies to ensure ease of administration and adequate pharmacokinetic properties, since no available treatments stop or reverse the progression of the disease. As of 2016, more than 1000 clinical trials have been or are being conducted to find ways to treat the disease, but it is unknown if any of the tested treatments will work.

## Author contributions

PB, PP, BP, FA, and VK write the study.

### Conflict of interest statement

The authors declare that the research was conducted in the absence of any commercial or financial relationships that could be construed as a potential conflict of interest.
